# Anxiety and hypertension in young and middle-aged adults: a longitudinal cohort study

**DOI:** 10.1093/pubmed/fdaf039

**Published:** 2025-04-10

**Authors:** Leah Jones, Jamie L Romeiser

**Affiliations:** Department of Public Health and Preventive Medicine, SUNY Upstate Medical University, 766 Irving Ave, Syracuse, NY, 13210, USA; Department of Public Health and Preventive Medicine, SUNY Upstate Medical University, 766 Irving Ave, Syracuse, NY, 13210, USA

**Keywords:** anxiety, hypertension, longitudinal studies, young adult

## Abstract

**Background:**

There is a significant prevalence of hypertension among younger and middle-aged adults. Anxiety is a potential psychosocial risk factor, but there is mixed evidence regarding this association in younger adults.

**Methods:**

This study analyzed data from the National Longitudinal Study of Adolescent to Adult Health, focusing on Waves 4 and 5, to explore the relationship between anxiety and incident hypertension in 9283 participants. Incident hypertension was classified first with objective blood pressure measures and antihypertensive medication use and, secondly, including self-reported diagnosis. Anxiety was identified in Waves 4 and 5, including age at diagnosis. Analyses used multivariable logistic regression and Cox proportional hazards models with anxiety treated as a time-dependent predictor.

**Results:**

The average age was ~29 at Wave 4 and ~37 at Wave 5. Results showed mixed evidence, with no significant association between prior anxiety and objectively measured hypertension. However, including self-reported hypertension, a prior diagnosis of anxiety was associated with incident hypertension (aOR = 0.76, 95% CI: 0.63–0.92). Time-to-event analyses further supported this inverse relationship.

**Conclusion:**

This study found some evidence suggesting an inverse relationship between anxiety and incident hypertension in young to middle-aged adults, warranting further longitudinal research into the complex relationship between mental and cardiovascular health.

## Introduction

An estimated 1.28 billion adults aged 30–79 globally have hypertension.^1^ Current guidelines in the USA recommend annual screening for both individuals 40 years or older and for younger individuals at an increased risk of hypertension.^2^ However, the prevalence of hypertension for young adults (aged 18–39) in the USA was 22.4% and 54.5% for middle-aged adults (aged 40–59) in 2017–18.^3^ Evidence has suggested that younger adults struggle to receive a timely diagnosis of hypertension compared to older adults.^4^ Undiagnosed and untreated hypertension at a younger age may cause health issues later in life such as cardiovascular disease, atherosclerosis, and problems in the endocrine system.^5^^,^^6^

There are various risk factors that have been found to potentially contribute to the development of hypertension, including different psychosocial factors. These factors may also affect how well a person’s hypertension is controlled.^7–9^ Anxiety disorders may be linked to the development of hypertension due to the physiological responses of chronic stress.^7^ Chronic exposure to stressors can cause disruptions to the neuroendocrine and immunological mechanisms of the body.^7^ These disruptions can result in the development of progression of physiological issues, including hypertension.^7^ Moreover, anxiety and other mental health issues may lead to uncontrolled hypertension resulting from unhealthy lifestyles, noncompliance with treatment plans, and underdiagnosis.^7–10^

While much of the existing research has focused on the relationship between anxiety and hypertension in older adults or populations with large age distributions, fewer studies have examined this relationship in younger and middle-aged adults.^11–20^ Some evidence suggests positive associations between anxiety and hypertension,^11–15^ though most of these studies are cross-sectional. Others have reported no associations or even an inverse relationship.^16–20^ Many of these studies utilize diverse methodologies for classifying and defining anxiety and hypertension, which may contribute to varying results.

The current body of evidence on young and middle-aged adults remains inconclusive. Given that high blood pressure (BP) often presents without any signs or symptoms,^21^ it remains important to thoroughly explore psychosocial risk factors that might influence its development in order to enhance screening protocols for younger populations. Therefore, this study adopts a multimodal approach to classify exposure and outcome variables, aiming to deepen our understanding of the nature of this relationship. The aim of this study is to investigate if there is an association between experiencing anxiety at younger ages and development of hypertension in young or middle-aged adulthood.

## Methods

Exemption status was received from SUNY Upstate Medical University’s Internal Review Board (#2145406-1).

### Study sample

This study used data from the National Longitudinal Study of Adolescent to Adult Health (Add Health), a nationally representative cohort study that collects longitudinal data from recruited adolescents in schools in the USA and follows them through young adulthood. At the time of this analysis, a total of five waves have been collected, spanning from 1994 to 2018. Anxiety was not assessed until Wave 4; therefore, this analysis focuses on data from Waves 4 and 5. Wave 4 (2008–09) consisted of 15 701 participants,^22^ with an average participant age of 28.9 years. Wave 5 (2016–18) included 12 300 participants, with an average participant age of 37.4 years.

We included participants who responded to health history questions (specifically regarding hypertension and anxiety) in Waves 4 and 5 (*n* = 10 854). We excluded participants with a prior self-reported diagnosis of hypertension at Waves 3 and 4 (*n* = 1389). Finally, because hypertension age at diagnosis was also assessed at the Wave 5 timepoint, we excluded any remaining participants who reported a diagnosis at an age prior to their age at Wave 4 or that occurred prior to any anxiety diagnosis (*n* = 182).

### Main outcome: hypertension

Systolic and diastolic blood pressures (SBP and DBP) were measured three separate times at Wave 5. As per published guidelines on the Wave 5 cardiovascular data,^23^ SBP was averaged using Measures 2 and 3. When Measure 2 or 3 was missing, Measure 1 was used to calculate the average. If Measures 2 and 3 were both missing, Measure 1 was used. The same methodology was used for the DBP. Average BP measurements were then classified using the Guideline for the Prevention, Detection, Evaluation, and Management of High Blood Pressure in Adults from the American Heart Association/American College of Cardiology Task Force (AHA/ACC) as normal (SBP < 120 mmHg and DBP < 80 mmHg), elevated (SBP: 120–129 mmHg and DBP: <80 mmHg), hypertension Stage 1 (SBP: 130–139 mmHg or DBP: 80–89 mmHg), Stage 2 (SBP: 140–180 mmHg or DBP: 90–120 mmHg), and crisis (SBP: >180 mmHg and DBP: >120 mmHg).^23^ Antihypertensive medication use was assessed in Wave 5. Furthermore, self-reported hypertension at Wave 5 was assessed as: “Has a doctor, nurse or other health care provider has ever told them that have high blood pressure or hypertension?”, with females being asked this question about the times they were not pregnant. Age of hypertension diagnosis was assessed by asking the following question: “How old were you when you were diagnosed by a doctor, nurse or other health care provider with high blood pressure or hypertension?”.

We used two approaches to operationalize hypertension. First, individuals were categorized as having hypertension (Hypertension 1) if their averaged BP was classified as Stage 1 or higher, or if they reported taking antihypertensive medication at Wave 5. Second, we expanded the definition (Hypertension 2) to include all individuals identified in Hypertension 1, as well as those who self-reported hypertension at Wave 5 but were not captured by Hypertension 1 (e.g. individuals who were not on medication or did not have elevated measured BP at that time point). To clarify, Hypertension 1 focuses on objective clinical measures and ensures that cases are based on standardized criteria. Hypertension 2 adds a somewhat subjective measure by including those who self-reported their diagnosis but perhaps do not meet clinical criteria due to effective management in the past. This allows for greater sensitivity in defining cases and attempts to capture what may have occurred between Waves 4 and 5. Time from Wave 4 to development of hypertension was also calculated using ages at Waves 4 and 5, as well as age at diagnosis. If individuals did not have an age at diagnosis but had measured BP at Stage 1 or above at Wave 5, the participant’s age at Wave 5 was used for the time-to-event analysis.

### Main exposure: anxiety

Anxiety was assessed in both Wave 4 and Wave 5 by asking participants: “Has a doctor, nurse, or other health care provider ever told you that you have or had anxiety or panic disorder?”. Additionally, age of anxiety diagnosis was assessed by asking the participants the following question in Wave 5: “How old were you when you were diagnosed by a doctor, nurse or other health care provider with anxiety or panic disorder?”.

We utilized the age of diagnosis for both anxiety and hypertension, along with participants’ ages at Waves 4 and 5, to operationalize two separate anxiety variables. The first approach classified anxiety as a prior condition (Prior Anxiety), which identified participants who reported a diagnosis of anxiety at Wave 4 or between Waves 4 and 5, provided that the age at anxiety diagnosis occurred before the age of any diagnosis of hypertension. This allowed us to explore the temporal relationship between anxiety and hypertension, focusing on anxiety as a predisposing factor for hypertension. The second approach expanded this classification to include both prior and concurrent diagnoses of anxiety (Prior or Concurrent Anxiety). This definition included participants whose reported age at diagnosis of anxiety coincided with the age of hypertension diagnosis between Waves 4 and 5 or those presenting with both conditions at the Wave 5 assessment. This broader definition was explored to allow for the possibility that anxiety and hypertension could have developed concurrently or perhaps that anxiety was undiagnosed prior to that time point.

### Covariates

Based on prior literature^24^^,^^25^ as well as American Heart Association guidelines^26^ we adjusted for numerous covariates from Wave 5 including sex, race, and ethnicity (non-Hispanic White, non-Hispanic Black/African American, Asian American or Pacific Islander, Hispanic, and Other), BMI (under or healthy weight [≤24.9], overweight [25.0–29.0] and obese (≥30.0])^27^, insurance status (private, Medicaid, Medicare, or uninsured), general health status (self-categorized as excellent/very good, good, and fair/poor), education level (less than high school, high school, some college, and college graduate or higher), diabetes, depression, and age. Frequency of sweetened beverage consumption, frequency of alcohol consumption, and smoking status were also included. Participants were asked “In the past 7 days, how many regular (non-diet) sweetened drinks did you have? Include regular soda, juice drinks, sweetened tea or coffee, energy drinks, flavored water, or other sweetened drinks,” which was dichotomized into not frequently (<7 times per week) and frequently (≥7 times per week). Frequency of alcohol consumption was dichotomized into not frequently (≤1–2 days a week) and frequently (3+ days per week). Smoking status was classified as a never smoker or any smoking by using three variables that asked participants “Have you ever smoked cigarettes regularly--that is, at least one cigarette every day for 30 days?”, “During the past 30 days, on how many days did you smoke cigarettes?”, and “During the past 30 days, on the days you smoked, how many cigarettes did you smoke each day?”.

### Statistical analysis

Multilevel clustering sample weights were applied to all analyses. Categorical variables are reported as frequencies and percentages, and continuous variables are reported as means and standard errors. Chi-squares were used to determine bivariate associations. Multivariable logistic regression was conducted to adjust for all covariates listed above. Four adjusted models explored the association between anxiety and hypertension: Models 1 and 2 examined the association between a prior diagnosis of anxiety and incident hypertension, with hypertension in Model 1 defined by high BP and/or use of antihypertensive medications, whereas hypertension in Model 2 also included a self-report of hypertension. Models 3 and 4 examined a prior or concurrent diagnosis of Anxiety and Hypertension 1 and 2, respectively. Finally, two-time dependent Cox proportional hazards models were also conducted, with anxiety as a time-varying predictor and all other variables as fixed covariates of hypertension. Analyses were conducted using SAS 9.4 © (Cary, NC) at the *P* = .05 significance level.

## Results

A total of 9283 met the inclusion criteria. Distribution of demographic and comorbid conditions are reported in Table 1. A total of 51.4% were females, 70.8% were White, and 55.0% had an education level of a college graduate or higher. Most reported having private insurance (78.4%). Overall, 47.9% reported being current smokers or having a history of smoking, 38.1% were obese, while most of the participants reported their general health as excellent or very good (54.7%) (Table 1). A total of 21.0% reported a diagnosis of anxiety prior to any diagnosis of hypertension, and an additional 0.9% had a prior or concurrent diagnosis of anxiety (21.9%). Twenty-one point seven percent had high BP or were on antihypertensive medications, and an additional 7.8% self-reported a diagnosis of hypertension (29.5%).

**Table 1:** Demographic and clinical characteristics of the National Longitudinal Study of Adolescent to Adult Health Sample (*n* = 9283).

Total sample		Hypertension 1 (measured BP or antihypertensive medication)			Hypertension 2 (including incident self-reported hypertension diagnosis)		
Characteristic	*N*(%)^a^	Yes *n*(%)^a^	No *n*(%)^a^	*P*-value^c^	Yes *n*(%)^a^	No *n*(%)^a^	*P*-value^c^
Prior anxiety diagnosis							
No	7433 (79.0)	1571 (78.8)	5862 (79.1)	.82	2170 (79.7)	5263 (78.7)	.48
Yes	1850 (21.0)	409 (21.2)	1441 (20.9)		509 (20.3)	1341 (21.3)	
Prior or concurrent anxiety diagnosis							
No	7362 (78.1)	1546 (77.4)	5816 (78.3)	.47	2099 (76.5)	5263 (78.7)	.09
Yes	1921 (21.9)	434 (22.6)	1487 (21.7)		580 (23.5)	1341 (21.3)	
Sex							
Male	3824 (49.3)	1033 (58.0)	2791 (46.9)	<.0001	1408 (59.7)	2416 (44.9)	<.0001
Female	5459 (50.7)	947 (42.0)	4512 (53.1)		1271 (40.3)	4188 (55.1)	
Insurance							
Insured	7524 (78.6)	1572 (77.6)	5952 (78.8)	.38	2106 (76.4)	5418 (79.3)	.0007
Medicaid	774 (9.1)	180 (9.8)	594 (8.9)		247 (9.6)	527 (8.9)	
Medicare	213 (2.8)	59 (3.5)	154 (2.6)		93 (4.3)	120 (2.2)	
Uninsured	731 (9.5)	161 (9.1)	570 (9.7)		222 (9.4)	509 (9.6)	
Alcohol consumption							
Frequently (3–5/week–everyday)	1555 (18.8)	392 (21.4)	1163 (81.9)	.02	519 (21.1)	1036 (17.9)	.006
Not frequently (0–1 or 2/week	7090 (81.2)	1460 (78.6)	5630 (18.1)		1981 (78.9)	5109 (82.1)	
Sweetened beverage consumption							
Frequently (≥7/week)	3582 (42.1)	864 (47.0)	2718 (59.3)	.0002	1160 (46.1)	2422 (40.3)	.0003
Not frequently (0–1 or 2/week)	5426 (57.9)	1089 (53.0)	4337 (40.7)		1461 (53.9)	3965 (59.7)	
Smoking frequency							
Any smoking	3778 (47.9)	857 (50.8)	2921 (47.1)	.04	1184 (51.3)	2594 (46.5)	.004
Nonsmoker	5275 (52.1)	1072 (49.2)	4203 (52.9)		1420 (48.7)	3855 (53.5)	
BMI							
Underweight or healthy weight	2824 (29.7)	335 (16.1)	2489 (33.5)	<.0001	428 (15.3)	2396 (35.7)	<.0001
Overweight	2981 (32.7)	645 (32.0)	2336 (32.9)		840 (31.1)	2141 (33.3)	
Obese	3434 (37.6)	991 (51.9)	2442 (33.6)		1398 (53.5)	2036 (30.9)	
General health							
Excellent or very good	5205 (54.8)	962 (48.4)	4243 (56.6)	<.0001	1170 (43.9)	4035 (59.4)	<.0001
Good	2984 (32.3)	729 (35.9)	2255 (31.2)		1045 (37.3)	1939 (30.1)	
Fair or poor	1080 (12.9)	288 (15.6)	792 (12.2)		463 (18.8)	617 (10.5)	
Race							
White	5704 (70.6)	1286 (73.3)	4418 (69.9)	<.0001	1663 (71.1)	4041 (70.5)	<.0001
Black	1748 (14.6)	420 (16.7)	1328 (14.0)		602 (17.4)	1146 (13.4)	
Asian American or Pacific Islander	566 (4.0)	81 (2.0)	485 (2.5)		127 (2.6)	439 (4.6)	
Hispanic	1129 (9.8)	171 (6.9)	958 (10.5)		248 (7.6)	881 (10.6)	
American Indian, Alaskan Indian, or other	103 (1.1)	19 (1.1)	84 (1.0)		31 (1.3)	72 (1.0)	
Education level							
Less than high school	336 (4.9)	73 (4.5)	263 (5.1)	.55	113 (4.8)	223 (5.0)	.03
High school	1275 (15.6)	269 (15.3)	1006 (15.7)		387 (16.0)	888 (15.5)	
Some college	2179 (25.1)	496 (26.9)	1701 (24.7)		698 (27.9)	1499 (23.0)	
College graduate or higher	5458 (54.3)	1138 (53.3)	4320 (54.6)		1476 (51.3)	3982 (55.5)	
Self-reported depression							
Yes	2239 (24.4)	548 (28.4)	1691 (24.6)	.02	749 (29.3)	1490 (23.8)	.0003
No	7015 (74.6)	1427 (71.6)	5588 (75.4)		1924 (70.7)	5091 (76.2)	
Self-reported diabetes diagnosis							
Yes	456 (4.9)	149 (7.6)	307 (4.1)	<.0001	240 (9.3)	216 (3.0)	<.0001
No	8798 (95.1)	1829 (92.4)	6969 (95.9)		2436 (90.7)	6362 (97.0)	
Age (mean, standard error)^b^	37.4 (0.10)	37.4 (0.10)			37.4 (0.10)		

a Weighted percent.

b Weighted mean and standard error.

c Chi-square analysis *P*-value results.

Assessed longitudinally, 21.2% of those who had measured hypertension (via BP or antihypertensive medication) had an anxiety diagnosis prior to incident hypertension. Using the expanded definition of hypertension including self-reported hypertension diagnosis, 20.3% had a prior or concurrent diagnosis of anxiety. Bivariate analyses found no significant association between a prior anxiety diagnosis and either hypertension outcome (*P* = .82, *P* = .48, respectively) (Table 1).

Including concurrent anxiety diagnoses, 22.6% of those who had measured hypertension had a prior or concurrent anxiety diagnosis at follow-up. Using the expanded definition of hypertension with self-diagnosis, 23.5% had a prior or concurrent diagnosis of anxiety. Bivariate analyses found no significant associations with either hypertension outcome (*P* = .47, *P* = .09, respectively) (Table 1).

After adjusting for the various demographic and health covariates, a prior diagnosis of anxiety showed decreased but nonsignificant odds of objective hypertension compared to those with no prior diagnosis, (Model 1, aOR 0.92 [95% CI 0.78–1.08], *P* = 0.31) (Table 2). However, in the expanded definition model (Model 2), a prior diagnosis of anxiety was significantly associated with decreased odds of incident hypertension (aOR = 0.76 [95% CI 0.63–0.92], *P* = .006) (Table 2).

**Table 2:** Multivariable logistic regression evaluating a prior anxiety diagnosis and hypertension.

	Model 1^a^			Model 2^b^		
Characteristic	OR	95% CI	*P*-value	OR	95% CI	*P*-value
Prior anxiety diagnosis	0.92	0.78–1.08	.31	0.76	0.63–0.92	.006
Sex						
Male	Reference	Reference	Reference	Reference	Reference	Reference
Female	0.64	0.54–0.75	<.0001	0.53	0.46–0.62	<.0001
Insurance status						
Insured	1.02	0.75–1.39	.90	1.02	0.75–1.38	.90
Medicaid	1.35	0.91–2.02	.14	1.21	0.81–1.81	.35
Medicare	1.40	0.86–2.29	.18	1.81	1.20–2.72	.005
Uninsured	Reference	Reference	Reference	Reference	Reference	Reference
Alcohol consumption						
Frequently (3–5/week–everyday)	1.36	1.14–1.64	.001	1.44	1.22–1.70	<.0001
Not frequently (0–1 or 2/week	Reference	Reference	Reference	Reference	Reference	Reference
Sweetened beverage consumption						
Frequently (≥7/week)	1.26	1.09–1.46	.003	1.21	1.05–1.40	.009
Not frequently (0–1 or 2/week)	Reference	Reference	Reference	Reference	Reference	Reference
Smoking status						
Any smoking	1.05	0.90–1.24	.52	1.09	0.03–1.29	.28
Nonsmoker	Reference	Reference	Reference	Reference	Reference	Reference
BMI category						
Underweight or healthy	Reference	Reference	Reference	Reference	Reference	Reference
Overweight	1.99	1.59–2.50	<.0001	2.12	1.72–2.61	<.0001
Obese	3.3	2.63–4.13	<.0001	3.97	3.25–4.84	<.0001
General health status						
Excellent or very good	Reference	Reference	Reference	Reference	Reference	Reference
Good	0.99	0.82–1.19	.92	1.16	1.0–1.96	.06
Fair or poor	1.05	0.80–1.37	.74	1.51	1.17–1.95	.002
Education status						
Less than high school	Reference	Reference	Reference	Reference	Reference	Reference
High school	1.29	0.82–2.05	.27	1.3	0.86–1.96	.21
Some college	1.46	0.93–2.30	.10	1.55	1.04–2.29	.03
College graduate or higher	1.6	1.01–2.53	.05	1.63	1.09–2.42	.02
Race						
White	Reference	Reference	Reference	Reference	Reference	Reference
Black	1.07	0.89–1.30	.47	1.17	0.99–1.40	.07
Asian American or Pacific Islander	0.56	0.35–0.88	.01	0.74	0.51–1.07	.11
Hispanic	0.58	0.41–0.83	.003	0.65	0.47–0.90	.009
Native American, Alaskan Native, or other	0.97	0.40–2.3	.94	1.23	0.64–2.34	.54
Self-reported depression diagnosis						
No	Reference	Reference	Reference	Reference	Reference	Reference
Yes	1.22	1.0–1.49	.05	1.46	1.19–1.78	.0003
Self-reported diabetes diagnosis						
No	Reference	Reference	Reference	Reference	Reference	Reference
Yes	1.59	1.13–2.24	.008	2.54	1.83–3.53	<.0001
Age	1.00	0.96–1.04	.94	1.01	0.97–1.05	.68

a Multivariable logistic regression of prior anxiety diagnosis and hypertension (measured BP or antihypertensive medication).

b Multivariable logistic regression of prior anxiety diagnosis and hypertension (including self-reported hypertension).

Additional adjusted models that examined a prior or concurrent anxiety diagnosis are presented in Table 3. Both models found decreased odds of hypertension for those with a prior or concurrent diagnosis of anxiety compared to those without, but relationships were not statistically significant (Table 3).

**Table 3:** Multivariable logistic regression evaluating a prior or concurrent anxiety diagnosis and hypertension.

	Model 1^a^			Model 2^b^		
Characteristic	OR	95% CI	*P*-value	OR	95% CI	*P*-value
Prior or concurrent anxiety diagnosis	0.96	0.81–1.12	.58	0.96	0.80–1.16	.68
Sex						
Male	Reference	Reference	Reference	Reference	Reference	Reference
Female	0.64	0.54–0.75	<.0001	0.52	0.45–0.61	<.0001
Insurance status						
Insured	1.02	0.75–1.39	.89	1.02	0.76–1.39	.88
Medicaid	1.35	0.91–2.02	.14	1.2	0.80–1.80	.38
Medicare	1.39	0.90–2.01	.18	1.75	1.16–2.64	.008
Uninsured	Reference	Reference	Reference	Reference	Reference	Reference
Alcohol consumption						
Frequently (3–5/week–everyday)	1.36	1.14–1.64	.001	1.44	1.22–1.70	<.0001
Not frequently (0–1 or 2/week	Reference	Reference	Reference	Reference	Reference	Reference
Sweetened beverage consumption						
Frequently (≥7/week)	1.26	1.09–1.46	.003	1.22	1.05–1.41	.009
Not frequently (0–1 or 2/week)	Reference	Reference	Reference	Reference	Reference	Reference
Smoking status						
Any smoking	1.05	0.90–1.24	.53	1.08	0.92–1.27	.33
Nonsmoker	Reference	Reference	Reference	Reference	Reference	Reference
BMI category						
Underweight or healthy	Reference	Reference	Reference	Reference	Reference	Reference
Overweight	2.00	1.59–2.50	<.0001	2.12	1.72–2.62	<.0001
Obese	3.30	2.63–4.13	<.0001	3.97	3.26–4.84	<.0001
General health status						
Excellent or very good	Reference	Reference	Reference	Reference	Reference	Reference
Good	0.99	0.82–1.19	.92	1.15	1.0–1.34	.06
Fair or poor	1.05	0.80–1.37	.74	1.5	1.16–1.93	.002
Education status						
Less than high school	Reference	Reference	Reference	Reference	Reference	Reference
High school	1.29	0.82–2.05	.27	1.3	0.87–1.96	.2
Some college	1.46	0.93–2.30	.10	1.54	1.01–2.28	.03
College graduate or higher	1.6	1.01–2.52	.05	1.62	1.09–2.4	.02
Race						
White	Reference	Reference	Reference	Reference	Reference	Reference
Black	1.08	0.89–1.30	.46	1.2	1	.05
Asian American or Pacific Islander	0.56	0.35–0.88	.01	0.76	0.52–1.1	.15
Hispanic	0.58	0.41–0.83	.003	0.66	0.48–0.90	.01
Native American, Alaskan Native, or Other	0.97	0.40–2.35	.94	1.22	0.64–2.33	.55
Self-reported depression diagnosis						
No	Reference	Reference	Reference	Reference	Reference	Reference
Yes	1.2	0.98–1.47	.07	1.32	1.08–1.62	.007
Self-reported diabetes diagnosis						
No	Reference	Reference	Reference	Reference	Reference	Reference
Yes	1.59	1.13–2.24	.008	2.52	1.82–3.50	<.0001
Age	1.00	0.96–1.04	.94	1.01	0.97–1.05	.67

a Multivariable logistic regression of prior or concurrent anxiety diagnosis and hypertension (measured BP or antihypertensive medication).

b Multivariable logistic regression of prior or concurrent anxiety diagnosis and hypertension (including self-reported hypertension).

Two exploratory models were run to examine time to incident hypertension from baseline age (age at Wave 4). The first model examined time to high BP or use of antihypertensive medications (Hypertension 1). After controlling for all covariates, at any given time point, individuals with anxiety had a lower risk of developing hypertension compared to those who did not have anxiety (hazard ratio [HR]: 0.74, [95% CI: 0.62, 0.87], *P* = .0003). Similar results were found when examining time to hypertension including those with self-reported diagnosis (Hypertension 2). After controlling for all covariates, at any given time point, individuals with anxiety had a lower risk of developing hypertension compared to those who did not have anxiety (HR: 0.72, [95% CI: 0.62, 0.83], *P* < .0001).

## Discussion

### Main findings

We examined the longitudinal association of anxiety and incident hypertension in young and middle-aged adults, finding mixed results. While three of our four models found no association, we did identify a significant negative association between a prior anxiety diagnosis and the more broadly defined hypertension outcome (Hypertension 1). Interestingly, time to incident hypertension analyses also suggested a significant protective effect of anxiety in this young adult cohort.

### What is already known

Much of the existing research on this relationship focuses on older populations or populations with larger age distributions^11–20^ with limited research focusing on younger to middle-aged adults. There appears to be mixed evidence regarding the relationship between anxiety and risk of hypertension in this population with existing research finding positive associations as well as negative or null associations.^11–20^ Existing studies appear to also use various methodologies in categorizing anxiety and hypertension variables.

### What this study adds

In general, our findings contrast with existing studies that found positive associations.^11–15^ Many of the studies that found positive associations assessed anxiety use various objective anxiety assessments while our study relied on self-reported anxiety diagnosis. Notwithstanding, the prevalence of anxiety in this study population is similar to nationally reported anxiety prevalence in similar age groups. The National Health and Nutrition Survey reports that ~20% of 18–29-year-olds and 17% of 30–44-year-olds experience symptoms of generalized anxiety disorder.^28^

Interestingly, some findings in our study are similar to a longitudinal study from Norway conducted in 2011,^16^ particularly our findings from the time-dependent Cox regression models. Hildrum *et al*. identified an inverse relationship between anxiety and incident hypertension over a 22-year period of time. There were, however, several differences. Our study used a self-reported diagnosis of anxiety, while Hildrum *et al*. measured anxiety through use of assessment scales. However, both used objective BP measurements to assess hypertension.

Additionally, our study found no significant association with either hypertension variable when anxiety was expanded to include both prior diagnosis and concurrent diagnoses. These findings corroborate other existing studies that were longitudinal or cross-sectional studies that also found no significant association.^17^^,^^18^^,^^20^

It’s possible that these inverse findings may involve both behavioral and physiological pathways. Individuals with anxiety disorders often utilize healthcare more due to a heightened awareness of symptoms.^29^ Increased health monitoring may lead to earlier detection or management of rising health issues such as elevated BP. Physiological factors, such as overexpression of neuropeptide Y (which may reduce BP) and fatigue/lower BP from anxiety induced hyperactivation, have also been proposed and described in detail elsewhere.^30^^,^^31^

A notable strength of our study is the inclusion of multiple strategies to capture hypertension, including self-reported diagnosis, measured BP, and antihypertensive medication use. While self-reports are useful in capturing subjective experiences but may be subject to recall bias, measured BP is more objective but may not account for the chronicity of hypertension. Combining these measures provided a more comprehensive assessment of hypertension. Furthermore, our study uses nationally representative data with both longitudinal and cross-sectional analyses, including several models to examine the association from multiple angles.

### Limitations

Our study has limitations. Certain covariates such as a comprehensive measure of physical activity or salt intake,^32^ were not assessed in the data, potentially resulting in residual confounding and inaccurate effect sizes. However, we did include numerous key risk factors, including sugar-sweetened beverage consumption, which may be a good indicator of diet quality.^33^^,^^34^ BP measurements were limited to Wave 5 only, which prevented analysis of change in average measurements over time. Our approach, while similar to some existing literature, may not fully capture the dynamic nature of BP and hypertension development, as well as limit the ability to determine a temporal relationship between anxiety and hypertension. Anxiety data were not collected prior to Wave 4, restricting our understanding of the impact of early-life anxiety and incident hypertension later in life. Finally, reliance on self-reported anxiety diagnosis and age of anxiety introduces the potential for misclassification and recall bias, which leads to under- or over-reporting and affects the study’s findings.

## Conclusions

Among younger to middle-aged adults without pre-existing hypertension, we found mixed evidence to suggest that those with a diagnosis of anxiety may have a reduced risk of hypertension in the future. Our mixed findings likely indicate that the relationship between anxiety and incident hypertension is complex and nuanced. Further exploration using verified clinical measures of anxiety, more frequent assessments of both anxiety and hypertension measures (e.g. blood pressure, medication assessments), and controlling for healthcare utilization patterns might help verify our findings. Further research should continue to investigate this relationship from a younger age to gain a comprehensive understanding of this relationship to further improve screening protocols for younger populations.

## Data Availability

The data underlying this article were provided by UNC Carolina Population Center under a restricted-use contractual agreement.

## Notes

**Leah Jones,** MPH, Student

**Jamie L. Romeiser,** PhD, MPH, Assistant Professor
